# Genomic, Trop‐2, and Immune Profiles of Carcinomatous and Sarcomatous Components in Ovarian Carcinosarcoma

**DOI:** 10.1002/cnr2.70659

**Published:** 2026-08-14

**Authors:** Tomomi Sakamaki, Tatsuyuki Chiyoda, Kohei Nakamura, Reika Takamatsu, Ryutaro Kawano, Daisuke Ochiai, Mitsuyo Jisaka, Mio Takahashi, Takuma Yoshimura, Yumiko Kimura, Shinya Oki, Kensuke Sakai, Tomoko Yoshihama, Hiroshi Nishihara, Wataru Yamagami

**Affiliations:** ^1^ Department of Obstetrics and Gynecology Keio University School of Medicine Tokyo Japan; ^2^ Center for Cancer Genomics, Keio University School of Medicine Tokyo Japan

**Keywords:** carcinosarcoma, CD8‐positive lymphocytes, epithelial‐mesenchymal transition, ovarian epithelial cancer, tumor immune evasion

## Abstract

**Background:**

Ovarian carcinosarcoma (OCS) is a rare, aggressive tumor composed of both carcinomatous and sarcomatous components, typically associated with poor prognosis. Due to its rarity, the molecular characteristics, immune microenvironment, and optimal treatment of OCS remain unclear. This study aimed to characterize the genomic profiles, Trop‐2 expression, and immune microenvironment of the carcinomatous and sarcomatous components of OCS separately, and to explore candidate therapeutic targets for this rare and aggressive tumor.

**Case:**

This retrospective, single‐institution study analyzed nine cases of ovarian or fallopian tube carcinosarcoma treated between 2013 and 2023. Targeted next‐generation sequencing of 160 cancer‐related genes was performed in eight cases, and immunohistochemistry for p53, Trop‐2, CD8, and PD‐L1 was performed in nine cases, with the carcinomatous and sarcomatous components evaluated separately whenever they were separable. All tumors contained high‐grade serous carcinoma components. Targeted sequencing revealed *TP53* mutations in all samples. Most genetic alterations were shared by both components, supporting the role of epithelial‐mesenchymal transition in OCS pathogenesis. Amplifications in *CCND1*, *CCNE1*, *PRKCI*, and *GNAS* (37.5%), and *ERBB2* (12.5%) were detected, and whole genome duplication was observed in two cases (25%). One case (12.5%) showed high microsatellite instability and was associated with Lynch syndrome. Immunohistochemistry showed Trop‐2 positivity in all carcinomatous components, with a trend toward higher expression compared with sarcomatous areas (*p* = 0.0625). CD8 and PD‐L1 levels were low, consistent with a relatively non‐inflamed, immune‐cold‐like microenvironment.

**Conclusion:**

These findings suggest molecular similarities between OCS and high‐grade serous carcinoma and highlight the role of epithelial‐mesenchymal transition. The low immune marker expression suggests limited benefit from immune checkpoint inhibitors, except in select Lynch syndrome cases. In contrast, the consistently high Trop‐2 expression in the carcinomatous component may warrant further investigation of Trop‐2‐targeted antibody–drug conjugates as a hypothesis‐generating therapeutic candidate for OCS.

**Abbreviations**
ADC: antibody‐drug conjugate
Bev: bevacizumab
CN‐H: copy number high
ddTC: dose‐dense paclitaxel + carboplatin
dMMR: deficient mismatch repair
EMT: epithelial‐mesenchymal transition
ESS: endometrial stromal sarcoma
HGSC: high‐grade serous carcinoma
HRD: homologous recombination deficiency
IDS: interval debulking surgery
MSI‐H: high microsatellite instability
OCS: ovarian carcinosarcoma
OS: overall survival
PARP: poly(adp‐ribose) polymerase
PARPi: PARP inhibitor
PDS: primary debulking surgery
PLD‐C: pegylated liposomal doxorubicin + carboplatin
RFS: recurrence free survival
SG: sacituzumab govitecan
TC: paclitaxel + carboplatin
TMB: tumor mutation burden
TMB‐H: high tumor mutation burden
VAF: variant allele frequency
WGD: whole genome duplication

## 1 Introduction

Ovarian carcinosarcoma (OCS) is a malignant ovarian tumor composed of carcinomatous and sarcomatous components and accounts for 1%–4% of all ovarian tumors [1]. It is reported to occur most often at around age 65 [2]. Although rare, it is more aggressive and has a poorer prognosis than conventional ovarian cancer, with a 5‐year survival rate of 26.63% for OCS compared to 43.61% for high‐grade serous carcinoma (HGSC) [3]. OCS is also rarer and has a poorer prognosis than uterine carcinosarcoma, with overall survival (OS) reportedly ranging from 8 to 26 months [4].

Molecular analysis of the carcinomatous and sarcomatous components suggests that carcinosarcomas do not develop from separate carcinoma and sarcoma origins, but rather the carcinoma transforms into sarcomatous components through epithelial‐mesenchymal transition (EMT) [5]. The epithelial component of OCS includes HGSCs, endometrioid carcinomas and undifferentiated carcinomas. Depending on the sarcomatous component, OCSs are further classified into homologous and heterologous subtypes. When the sarcomatous component is of Müllerian origin such as fibrosarcoma, endometrial stromal sarcoma, or leiomyosarcoma, it is classified as homologous. When differentiation into non‐Müllerian mesenchymal tissues such as chondrosarcoma, rhabdomyosarcoma, or liposarcoma is observed, it is classified as heterologous. Although carcinosarcoma is rare, OCS is even rarer than uterine carcinosarcoma, resulting in fewer reports on OCS and limited understanding of its clinical characteristics. In OCS, the majority of the carcinomatous component is HGSC, with less than 20% being endometrioid carcinoma [6]. OCS is thought to develop mainly through EMT of HGSC [7].

Regarding treatment for OCS, the same approach as for ovarian cancer is employed, consisting of tumor debulking surgery followed by platinum‐based combination chemotherapy, but due to its rarity, the effectiveness of treatment in OCS remains unclear. As OCS has a poor prognosis, novel treatment strategies are needed. Immune checkpoint inhibitors have been introduced for the treatment of uterine endometrial cancer. The effectiveness of immune checkpoint inhibitors varies depending on the status of DNA mismatch repair (MMR) proteins, but their efficacy in endometrial cancer has been demonstrated even with proficient MMR [7]. Although the Ruby trial of endometrial cancer included about 10% of carcinosarcoma cases, most carcinosarcomas of uterine carcinosarcomas are endometrioid carcinomas [8], suggesting that OCS (predominantly serous carcinoma) and uterine carcinosarcoma are molecularly distinct. It has been reported that 88% of OCS are copy number high (CN‐H) compared to 53% of uterine carcinosarcomas [8].

The strong efficacy of immune checkpoint inhibitors in ovarian cancer, especially HGSC, has not been demonstrated to date [9]. The therapeutic effect of immune checkpoint inhibitors on OCS is still unclear. Tumor immunosuppression not only promotes tumor formation but also inhibits anti‐tumor effects. PD‐L1 is a major regulatory factor of immunosuppression that is expressed on tumor‐infiltrating CD8+ T cells, CD4+ T cells, natural killer T cells, B cells, dendritic cells, and macrophages. It has been reported that 33% of carcinosarcomas are PD‐L1 positive/CD8 positive, but this report did not separate uterine carcinosarcoma from OCS [10]. A report on 19 OCS cases showed that PD‐L1 was positive in about 50% of both carcinomatous and sarcomatous components, and CD8+ T cells were positive in 37% of carcinomatous and 84% of sarcomatous components [11]. Although it has been suggested that CD8 positivity in the sarcomatous component indicates a better prognosis, reports are limited to a small number of cases, and the immune microenvironment of OCS remains largely unclear.

Human trophoblast cell‐surface marker (Trop‐2) is a monomeric transmembrane cell surface glycoprotein originally found in placental trophoblastic tissue. It is elevated in various cancers but has low expression in normal tissues [12]. In ovarian cancer, high protein expression of Trop‐2 has been reported as a poor prognostic factor [13]. Sacituzumab govitecan (SG, IMMU‐132, Gilead, Foster City, CA, USA) is an antibody‐drug conjugate (ADC) that combines a humanized Trop‐2 antibody with an active metabolite of irinotecan (SN‐38) [14]. In epithelial ovarian cancer, Trop‐2 moderate‐to‐strong staining was observed in 47% of cases, and SG showed anti‐tumor effects in chemotherapy‐resistant ovarian cancer xenografts [15]. Both uterine carcinosarcoma and OCS showed strong Trop‐2 expression in 30% of cases, and SG has been shown to be effective, showing promise in the treatment of carcinosarcoma [16].

Reports on genomic analysis of OCS are mainly limited to a small number of cases. A study that performed whole exome sequencing, micro RNA profiling, and mRNA profiling on both carcinomatous and sarcomatous components of 12 OCS cases found *CCNE1* amplification in 50%, loss of X chromosome in 67%, loss of chr8p in 42%, and copy number amplification of chr20 including *SRC* and *GNAS* in the sarcomatous component [17]. In bulk targeted sequencing of OCS, 5 out of 13 CN‐H cases (38%) had *MECOM* amplification, 3 cases (23%) had *MYC* amplification, and 2 cases (15%) had *CCNE1* amplification [8].

As mentioned above, while OCS has many molecular biological unknowns due to its rarity, it is a disease that requires treatment development because of its poor prognosis and intractability. Therefore, in this study, to better understand the clinical and pathological characteristics of OCS, we analyzed 9 cases from our institution, examining pathological features, immunohistochemical analysis of p53, Trop‐2, CD8, and PD‐L1, and molecular profiling through comprehensive genomic profiling using next‐generation targeted sequencing, separately for carcinomatous and sarcomatous components, and retrospectively analyzed these results with clinicopathological factors.

## 2 Materials and Methods

### 2.1 Study Design

This was a retrospective, single‐institution case series conducted at Keio University Hospital, Tokyo, Japan, covering the study period from 2013 to 2023. Patients were eligible for inclusion if they had a histologically confirmed diagnosis of ovarian or fallopian tube carcinosarcoma and were treated at our institution during this period. Cases were excluded if archival tumor material was insufficient for immunohistochemical or genomic analysis. The clinicopathological variables collected were age at initial treatment, FIGO stage, primary anatomic site, the histological subtypes of the carcinomatous and sarcomatous components, surgical approach and completeness of resection, adjuvant chemotherapy and maintenance therapy, recurrence, and survival status. The study endpoints were recurrence‐free survival (RFS) and overall survival (OS). Nine cases met these criteria and were analyzed clinically and pathologically. OS was defined as the time from initial surgery to death from any cause or last follow‐up. RFS was defined as the time from initial surgery to the first documented recurrence or death.

### 2.2 Immunohistochemistry

Immunohistochemical staining was performed for p53, PD‐L1, Trop‐2, and CD8 using respective antibodies. p53 staining was performed as previously described [18]. PD‐L1 expression was evaluated using the 28‐8 clone antibody (Abcam, Cambridge, UK, ab205921, 1:400) incubated overnight at 4°C, and Trop‐2 (Abcam, ab227691, 1:300) and CD8 (DAKO, Glostrup, DK, M7103, 1:150) were incubated for 60 min at room temperature. For all markers, antigen retrieval was performed at 95°C for 20 min using a high pH buffer (DAKO, K8004). Detection was performed using HistoFine Simple Stain MAX‐PO (Nichirei Bioscience, Tokyo, Japan), and for PD‐L1, a LINKER (DAKO) was added before DAB staining (Nichirei Bioscience) for 5 min. All IHC slides were evaluated by a board‐certified pathologist. The percentage of CD8‐positive cells among total lymphoid cells and PD‐L1‐positive cells among total lymphoid cells were recorded for each component. PD‐L1 expression in tumor cells was also assessed as the percentage of positively stained tumor cells. Trop‐2 expression was assessed as the percentage of positive tumor cells.

### 2.3 NGS‐Based Cancer Gene Panel Test

Rapid Neo was performed as a genomic profiling test for cancer gene panel analysis [19]. For CS3, the endometrioid and mucinous components were subjected to macrodissection, and for other samples, DNA extraction and genomic analysis were performed using respective slides. Rapid Neo is a next‐generation sequencing‐based multiplex gene assay corresponding to the PleSSision‐160 platform. In brief, frozen tissue samples were fixed with the PAXgene Tissue System (QIAGEN, Germantown, MD, USA) and paraffin‐embedded; a pathologist evaluated tumor cell content on hematoxylin–eosin–stained slides and performed macrodissection when necessary. Genomic DNA was extracted and purified, and DNA quality was assessed using the DNA integrity number (DIN) score on an Agilent 2200 TapeStation (Agilent Technologies), with libraries prepared only from DNA with a DIN score > 3.1. Targeted amplicon exome sequencing of 160 cancer‐related genes was performed on an Illumina MiSeq platform (Illumina, San Diego, CA, USA), and sequencing data were annotated and curated using the GenomeJack bioinformatics pipeline (Mitsubishi Space Software, Tokyo, Japan). Single‐nucleotide variants, insertions/deletions, and copy‐number variations were identified, and tumor mutation burden (TMB) and copy‐number alterations (CNAs) were derived from these data. For CNA calling, read counts per amplicon were normalized to reads per million and compared with a baseline established from at least 100 FFPE samples; using amplicons with a coefficient of variation < 0.32 and mean > 10, the per‐gene median log2 ratio (sample/baseline) was calculated, and genes with a median log2 ratio exceeding +1 SD or +2 SD were classified as amplification‐like or amplification, respectively (and below −SD or −2SD as loss‐like or loss). Fifty genomic DNA samples were used as controls to normalize read depth per amplicon; only amplicons with a depth CV ≤ 1.5 were used, and CNA was calculated for genes with more than six amplicons. Hypermutation was defined as ≥ 10 SNVs/Mb and ultramutation as ≥ 100 SNVs/Mb. Microsatellite instability was determined using MSIsensor, with a score ≥ 12 defining MSI‐high (MSI‐H), and the homologous recombination deficiency (HRD) score was calculated using an algorithm analogous to the loss‐of‐heterozygosity (LOH) score of the Myriad myChoice CDx assay (Myriad Genetics, Salt Lake City, UT, USA); because telomeric allelic imbalance and large‐scale state transitions cannot be reliably assessed on a targeted gene panel, the score was instead defined from genome‐wide copy‐number breakpoints, quantified as the percentage of detected breakpoints reflecting differences in CNA status (loss, neutral, or amplification) between adjacent probe genes. LOH regions spanning ≥ 90% of a whole chromosome or chromosome arm were regarded as arising from non‐HRD mechanisms and excluded; chromosomes with fewer than two probe genes (chromosomes 8, 18, 21, and X) and chromosomes with uniform CNA status were likewise excluded, with an HRD score > 10 considered high. All analyses were performed on tumor tissue only, without matched germline (normal) controls; germline variants were therefore excluded using algorithmic filtering and population‐frequency databases, which limits definitive discrimination between somatic and germline variants [20].

### 2.4 Statistical Analyses

Statistical analysis was performed using GraphPad Prism 7.04 (GraphPad Software, La Jolla, CA, USA) with Wilcoxon signed‐rank test (paired). For CS7 and CS9, in which the carcinomatous and sarcomatous components were inseparably admixed, these cases were excluded from the statistical analysis (*n* = 7 pairs). For CS3, in which three carcinomatous histologies were present, the serous component (listed as “carcinoma (serous)” in Table 2) was used as the carcinomatous value in the statistical analysis.

## 3 Results

### 3.1 Case Overview

The study included nine cases, with patient age at initial treatment ranging from 42 to 73 years, with a median of 63 years. In two cases, exploratory laparotomy was performed for abdominal observation and pathological diagnosis. In one case where the primary site was unknown, diagnosis was made by ultrasound‐guided lymph node biopsy. Neoadjuvant chemotherapy was performed in this case and in one case that underwent exploratory laparotomy. The other seven cases underwent primary debulking surgery (PDS). As a result of surgery, the primary site was the ovary in six cases and the fallopian tube in three cases. According to FIGO classification (FIGO 2014), there were two cases in stage I, one case in stage II, five cases in stage III, and one case in stage IV (Table 1). More than half were advanced cancers. In one case, stage IB endometrial cancer was found simultaneously.

**TABLE 1:** Clinicopathologic data of nine OCS cases.

	Age	Pathology			TNM	Surgical approach	Surgical completeness	Adjuvant chemotherapy	Maintenance therapy	Recurrence	RFS (months)	Death	OS (months)
		Epithelial	Sarcomatous	Primary anatomic site									
CS1^a^	44	HGSC	chondrosar. + rhabdomyosar.	O	pT1aN0M0	PDS	Complete	TC	−	+	15.9	−	91.5
CS2	64	HGSC	ESS + chondrosar.	T	pT3cN0M0	PDS	Complete	TC + Bev	Bev	+	13.6	+	34.8
CS3	63	HGSC + endometrioid ca. + mucinous ca.	Atypical stromal cells	O	pT1cN0M0	PDS	Complete	TC	−	−	46.7	−	47.7
CS4	42	HGSC + NEC	Chondrosar.	T	pT2bN0M0	PDS	Complete	TC	−	−	45.9	−	51.1
CS5	44	HGSC + endometrioid ca.	sar. (NOS)	O	pT3bN1aM0	PDS	Complete	PLD‐C	PARPi	+	39.2	−	45.7
CS6	69	HGSC	sar. (NOS)	O	pT1cN1M0	PDS	Complete	ddTC + Bev	Bev	+	9.8	+	53.5
CS7	60	HGSC	Rhabdomyosar.	O	ypT3cN0M1a	IDS	Complete	TC + Bev	PARPi + Bev	+	3	+	8.2
CS8	73	HGSC	Chondrosar.	O	ypT3bN0M0	IDS	Complete	TC + Bev	Bev	−	58.7	−	61.7
CS9	67	HGSC	Leiomyosar.	T	pT3bN1aM0	PDS	Complete	ddTC	−	+	17.1	+	70
Median (range)	63 (42–73)										17.1 (3.0–58.7)		51.1 (8.2–91.5)

Abbreviations: Bev: bevacizumab; ddTC: dose‐dense TC; ESS: endometrial stromal sarcoma; HGSC: high‐grade serous carcinoma; IDS: interval debulking surgery; NEC: neuroendocrine carcinoma; NOS: not otherwise specified; O: ovary; OS: overall survival; PARPi: PARP inhibitor; PDS: primary debulking surgery; PLD‐C: pegylated liposomal doxorubicin + carboplatin; RFS: recurrence free survival; T: fallopian tube; TC: paclitaxel + carboplatin.

^a^ Lynch syndrome.

One patient (CS1) was a Lynch syndrome patient who developed ovarian carcinosarcoma after fertility‐preserving therapy for endometrial cancer. All nine OCS cases had HGSC components in the carcinomatous component, and three cases had serous carcinoma coexisting with other histologies. One case had neuroendocrine carcinoma (CS4), one case had endometrioid carcinoma (CS5), and another case had both endometrioid and mucinous carcinoma (CS3). The sarcomatous component included chondrosarcoma in four cases (44%) and rhabdosarcoma in two cases (22%). Notably, in CS7 and CS9, the carcinomatous and sarcomatous components were intimately admixed without a clearly definable boundary.

Complete macroscopic tumor resection was achieved in all cases. Platinum‐based combination chemotherapy was administered in all cases, and maintenance therapy was provided in five cases (bevacizumab, three cases; poly(ADP‐ribose) polymerase (PARP) inhibitor, one case; PARP inhibitor + bevacizumab, one case). Recurrence was observed in six cases (66.7%), with a median recurrence‐free survival of 17.1 months (range 3.0–58.7), and four cases (44%) resulted in death, with a median OS of 51.1 months (range 8.2–91.5).

### 3.2 Many Genetic Mutations Are Common Between Carcinomatous and Sarcomatous Components, but Not All

**FIGURE 1:**
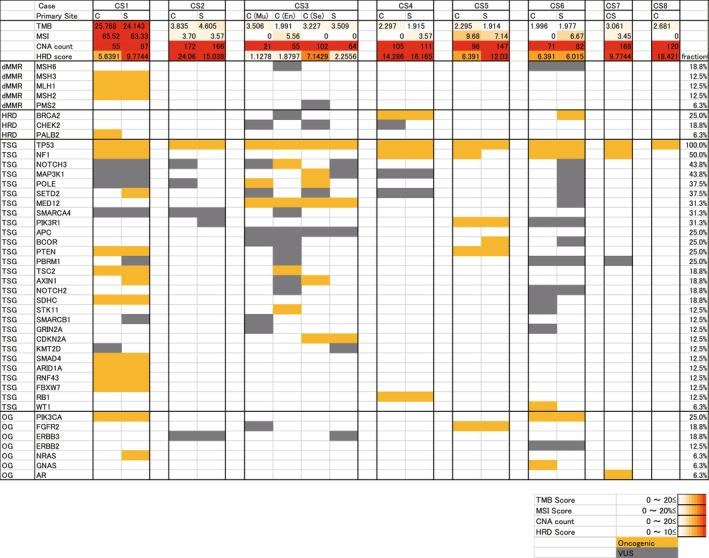
Actionable gene variants identified in ovarian carcinosarcoma. Oncogenic variant and variant of unknown significance (VUS) are depicted. In CS7, the carcinomatous and sarcomatous components were inseparably admixed and were sequenced in bulk; this tumor is shown as a single column labeled CS. In CS8, the sarcomatous component yielded DNA of insufficient concentration for sequencing, and only the carcinomatous component (C) is shown. C: carcinoma; CNA: copy number alteration; CS: carcinosarcoma; dMMR: deficient of mismatch repair; En: endometrioid; HRD: homologous recombination deficiency; Mu: mucinous; OG: oncogene; S: sarcoma; Se: serous; TMB: tumor mutation burden; TSG: tumor suppressor gene; VAF: variant allele frequency.

Genomic analysis by targeted sequencing was performed on 8 out of 9 cases (Figure 1). One case (CS9) could not undergo genomic analysis due to poor DNA quality. In CS7, the carcinomatous and sarcomatous components were inseparably admixed and the tumor was therefore sequenced in bulk, so all genomic values reported for CS7 (TMB, MSI score, CNA count, HRD score, and variant calls) represent the admixed tumor rather than the individual components. In CS8, the two components could be separated histologically, but the DNA recovered from the sarcomatous component was of insufficient concentration for library preparation; the genomic data reported for CS8 therefore derive from the carcinomatous component only. Component‐resolved data for both components were thus available in six cases (CS1‐CS6). For CS3, the carcinomatous component contained mucinous carcinoma, endometrioid carcinoma, and serous carcinoma, which were sequenced separately.

In the Lynch syndrome case CS1, both carcinomatous and sarcomatous components showed high TMB values of 25.7 and 24.1 mut/Mb, respectively, and both components also showed high microsatellite instability (MSI‐H). Mutations in *MSH3*, *MLH1*, *MSH2* were found in both carcinomatous and sarcomatous components. *TP53*, *NF1*, *PTEN*, *FBXW7*, *ARID1A*, and *PIK3CA* mutations were common to both components. *PALB2* mutations were found only in the carcinomatous component, while *SETD2*, *AXIN1*, and *NRAS* mutations were found only in the sarcomatous component (Figure 1). In CS2, *TP53* and *SMARCA4* (*BRG1*) mutations were common to both carcinomatous and sarcomatous components. In CS3, *TP53* and *MED12* mutations were common across all carcinomatous components (mucinous, endometrioid, and serous) and the sarcomatous component. *TSC2* mutations were found only in the endometrioid component, while *CDKN2A* mutations were common to both the serous carcinoma component and the sarcomatous component. In CS3, *STK11* mutations were specifically detected in the endometrioid carcinoma component. In CS4, *TP53*, *BRCA2*, and *NF1* mutations were common to both carcinomatous and sarcomatous components, and in CS5, *TP53*, *PIK3R1*, and *PTEN* mutations were common, but *BCOR* mutations were detected only in the sarcomatous component, and *NF1* mutations only in the carcinomatous component. In CS6, *TP53*, *NF1*, and *PIK3CA* mutations were common to both carcinomatous and sarcomatous components, but *BRCA2* mutations were found only in the sarcomatous component, and *GNAS* mutations only in the carcinomatous component. All 8 cases (100%) had *TP53* mutations, and only the Lynch syndrome case CS1 (12.5%) was TMB‐H and MSI‐H. An HRD score > 10 was observed in four cases (50%): in CS2 and CS4, both the carcinomatous and the sarcomatous components exceeded this threshold; in CS5, the threshold was exceeded in the sarcomatous component only; and in CS8 it was exceeded in the carcinomatous component, which was the only component that could be sequenced in that case (Figure 1).

### 3.3 DNA Copy Number Changes in Carcinomatous and Sarcomatous Components

**FIGURE 2:**
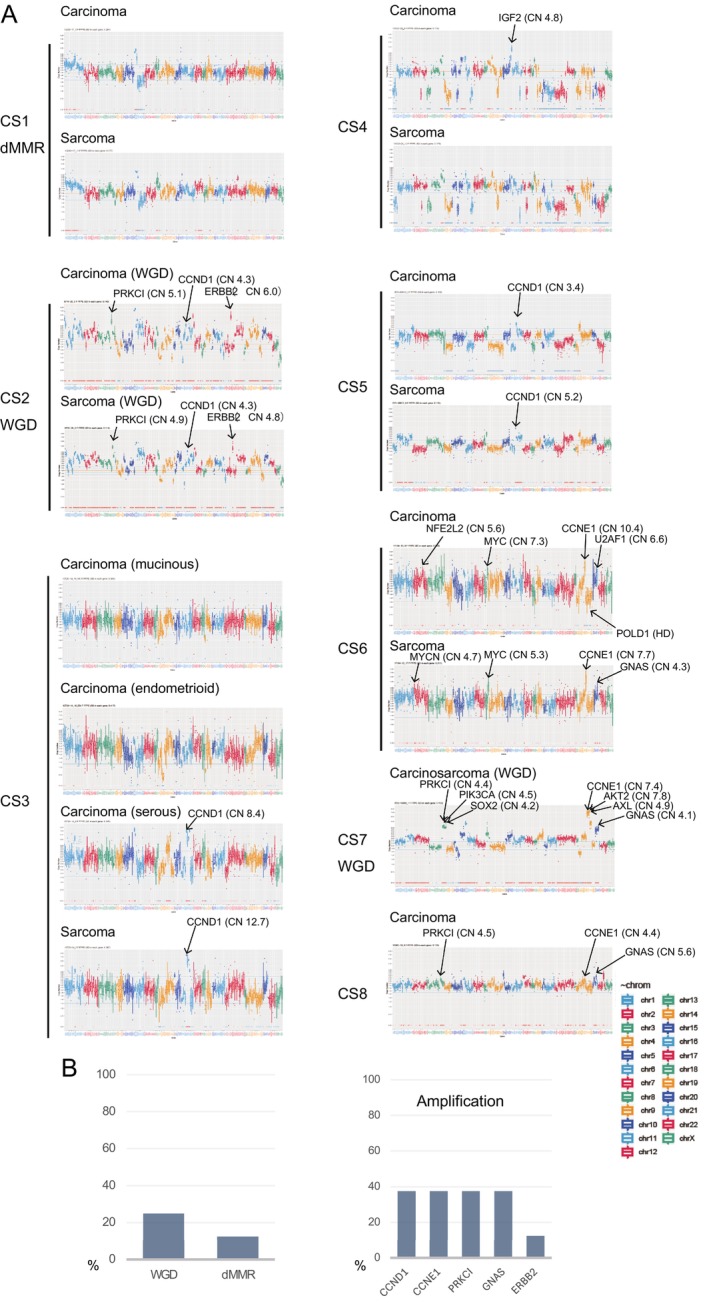
Copy‐number alterations in ovarian carcinosarcoma. (A) The horizontal axis corresponds to each examined gene, and the vertical axis corresponds to the copy number. (B) Frequencies of WGD, dMMR, and gene amplification in ovarian carcinosarcoma (*n* = 8 evaluable cases). WGD: 25% (2/8); dMMR: 12.5% (1/8); *CCND1* amplification: 37.5% (3/8); *CCNE1* amplification: 37.5% (3/8); *PRKCI* amplification: 37.5% (3/8); *GNAS* amplification: 37.5% (3/8); *ERBB2* amplification: 12.5% (1/8). dMMR: deficient DNA mismatch repair; WGD: whole genome duplication.

DNA copy number changes were also similar between carcinomatous and sarcomatous components (Figure 2). Two cases (25%), CS2 and CS7, showed whole genome duplication (WGD). *CCND1*, *CCNE1*, *PRKCI*, and *GNAS* amplifications were each found in 3 cases (37.5%), and *ERBB2* amplification in 1 case (12.5%). *GNAS* amplification was detected in the sarcomatous component of CS6, in the bulk‐sequenced tumor of CS7, and in the carcinomatous component of CS8. In CS3, *CCND1* amplification was observed in the serous carcinoma component and the sarcomatous component, but not in the mucinous or endometrioid components. In CS4, *IGF2* amplification was found only in the carcinomatous component.

### 3.4 Analysis of p53, Trop‐2, CD8, and PD‐L1 Expression in Carcinomatous and Sarcomatous Components

**FIGURE 3:**
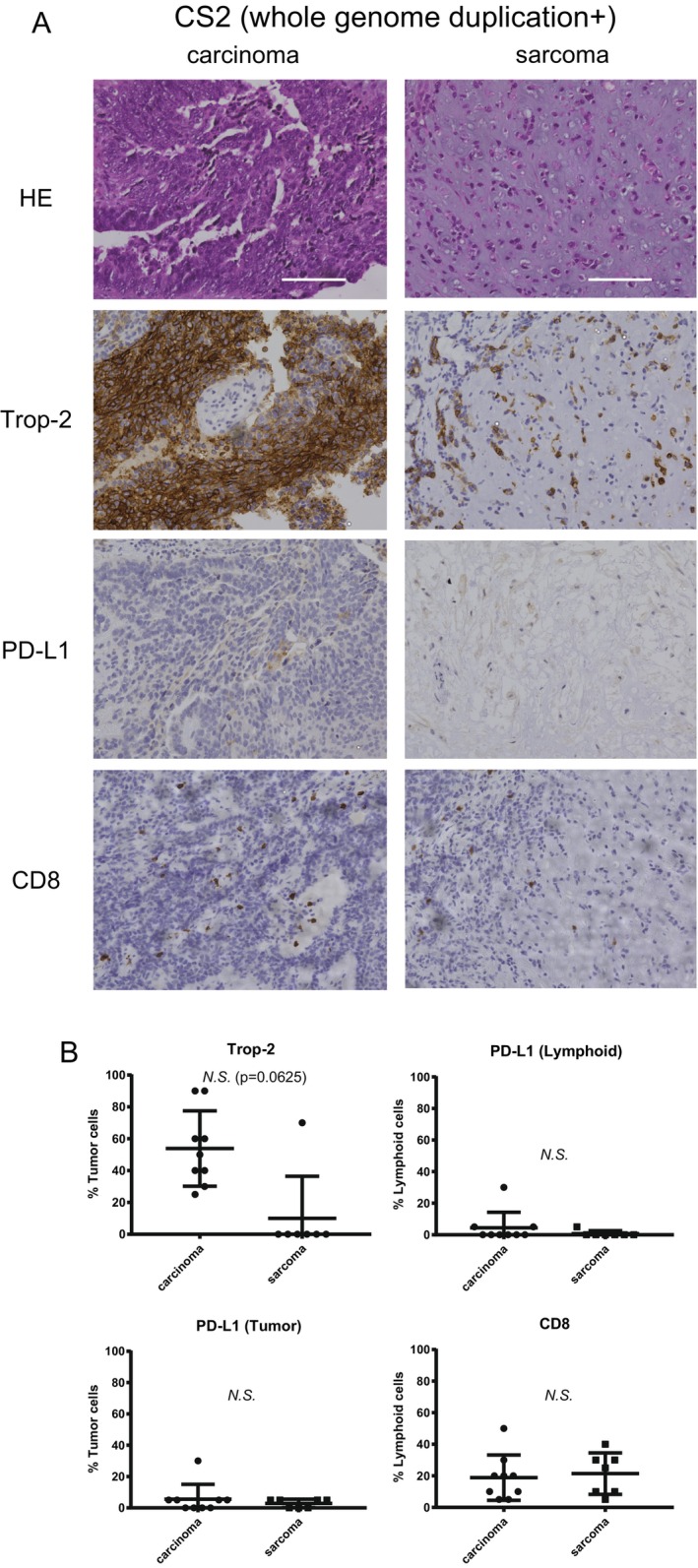
Trop‐2 showed high positivity in the carcinomatous component of ovarian carcinosarcoma. (A) Representative hematoxylin–eosin (HE), Trop‐2, PD‐L1, and CD8 immunohistochemistry of the carcinomatous and sarcomatous components in CS2 (whole genome duplication‐positive). Scale bars, 100 μm. (B) Trop‐2, PD‐L1 (lymphoid cells), PD‐L1 (tumor cells), and CD8 expression in carcinomatous and sarcomatous components of all 9 cases. CS7 and CS9, in which carcinomatous and sarcomatous components were inseparably admixed, are plotted in the carcinoma group but were excluded from statistical analysis (*n* = 7 pairs). Horizontal bars indicate mean ± SD. Statistical comparisons were performed using the Wilcoxon signed‐rank test. N.S., not significant.

Next, protein expression analysis of p53, Trop‐2, CD8, and PD‐L1 was performed on nine cases. Trop‐2, PD‐L1, and CD8 expression in the carcinomatous and sarcomatous components of all cases is summarized in Figure 3 and Table 2, and representative IHC images (HE, Trop‐2, PD‐L1, and CD8) are shown for CS2 in Figure 3A. Trop‐2 was positive in the carcinomatous component in all cases (100%), but only in the sarcomatous component in 1 case (11.1%). The positivity rate in the carcinomatous component was 25%–90% (median 50%), and in the sarcomatous component 0%–70% (median 0%), with a trend toward higher expression in the carcinomatous component, which did not reach statistical significance (*p* = 0.0625, Wilcoxon signed‐rank test, *n* = 7 pairs; CS7 and CS9 excluded). PD‐L1 expression in lymphoid cells was low in both carcinomatous (positivity rate 0%–30%, median 0%) and sarcomatous components (positivity rate 0%–5%, median 0%), with CS8 showing the highest tumor cell PD‐L1 positivity at 30% in the carcinomatous component. CD8 showed positivity rates of 5%–50% (median 20%) in the carcinomatous component and 5%–40% (median 25%) in the sarcomatous component, with low expression in both components. In p53 immunostaining, the Lynch syndrome case CS1 (*TP53* p.R181C) showed weak positivity for p53, displaying a wild‐type pattern. CS2 (*TP53* c.673‐2A>G) and CS4 (*TP53* c.672+2T>G), both with *TP53* splice site mutations, showed a null pattern for p53. The other six cases all showed p53 overexpression patterns.

**TABLE 2:** CD8, PD‐L1, Trop‐2 expression in carcinomatous and sarcomatous components of ovarian carcinosarcoma.

	Component	CD8 (% lymphoid cells)	PD‐L1 (% lymphoid cells)	PD‐L1 (% tumor cells)	Trop‐2 (% tumor)
CS1	Carcinoma	20	0	0	90
	Sarcoma	10	0	5	0
CS2	Carcinoma	10	0	5	40
	Sarcoma	30	5	5	70
CS3	Carcinoma (serous)	10	0	0	50
	Carcinoma (endometrioid)	20	0	0	10
	Carcinoma (mucinous)	10	0	0	10
	Sarcoma	40	0	0	0
CS4	Carcinoma	5	5	0	30
	Sarcoma	5	0	5	0
CS5	Carcinoma	20	0	5	90
	Sarcoma	30	0	5	0
CS6	Carcinoma	20	0	5	60
	Sarcoma	10	0	0	0
CS7	Carcinosarcoma	30	5	5	60
CS8	Carcinoma	50	30	30	25
	Sarcoma	25	0	0	0
CS9	Carcinosarcoma	5	0	0	40

## 4 Discussion

There are several reports on the prognosis of OCS, many of which indicate OS of generally less than 2 years [21]. OCS is considered to have the worst prognosis among all ovarian tumors, even compared to HGSC, which is generally considered to have a poor prognosis. OCS is also reported to have a worse prognosis than uterine carcinosarcoma [8]. In a study analyzing 22 cases of uterine carcinosarcoma, 14 cases of OCS, and 1 case of cervical carcinosarcoma, recurrences were more common in OCS cases where the carcinomatous component was HGSC [21].

Focusing on molecular classification, OCS has a higher frequency of CN‐H than uterine carcinosarcoma [8]. CN‐H is known to be the subtype with the worst prognosis among the four molecular classifications of endometrial cancer. CN‐H often has *TP53* mutations, which is consistent with our finding that all eight OCS cases with evaluable genomic data harbored *TP53* mutations. CS9 could not be sequenced because of poor DNA quality, and its *TP53* mutation status is therefore unknown, although p53 immunostaining in this case showed an overexpression pattern.

While there is no clear treatment strategy, appropriate tumor debulking surgery has been reported to improve prognosis. Advanced stage, sarcomatous component exceeding 25%, VEGF expression, onset before age 65, and *TP53* mutations are considered poor prognostic factors [22]. In this study, complete tumor resection was achieved in all cases. Our results showed a median OS of 51.1 months, which is better than previously reported, suggesting that multimodal treatment with maximum tumor reduction surgery and postoperative adjuvant chemotherapy/maintenance therapy may lead to a favorable prognosis. However, as complete macroscopic resection was achieved in all cases, selection bias cannot be excluded. Given the heterogeneity in disease stage and treatment regimens across cases, this finding should be interpreted with caution, and definitive attribution to any specific treatment modality is not possible.

Regarding chemotherapy, paclitaxel + carboplatin (TC therapy) for uterine carcinosarcoma has been shown to be non‐inferior to the combination of paclitaxel and ifosfamide and is considered the standard treatment [23]. However, due to the small number of cases in OCS, while TC therapy is commonly used for OCS as in uterine carcinosarcoma, evidence for its effectiveness is limited. In recent years, PD‐1/PD‐L1 inhibitors have shown therapeutic effects in various cancer types and have become standard treatments. Although strong therapeutic effects have not been demonstrated in ovarian cancer so far, progression‐free survival extension with the combination of PARP inhibitors, immune checkpoint inhibitors, and bevacizumab has been shown in ovarian cancer [24]. However, in a phase II trial using ipilimumab, a CTLA‐4 and PD‐1 double blockade, the response rate for 6 OCS cases was 0% [25]. In this study, the low CD8 and PD‐L1 expression is consistent with a predominantly immune‐cold phenotype in most cases, although this assessment was limited to two markers and warrants confirmation with a broader immune panel. However, in this study, 1 case (12.5%) of deficient MMR (dMMR) was identified, suggesting that it is necessary to investigate MMR status in advanced/recurrent OCS to determine the applicability of immune checkpoint inhibitors.

Trop‐2 is the target of SG, an ADC approved for the treatment of triple‐negative breast cancer and urothelial cancer. SG has shown preclinical therapeutic efficacy in uterine carcinosarcoma and OCS [26]. In this study, Trop‐2 was positive in the carcinomatous component in all cases, but expression was low in the sarcomatous component (Figure 3). SG has a bystander effect and exerts anti‐tumor effects on surrounding tissues [16]. Therefore, in OCS, the therapeutic effect of SG is expected to extend to the sarcomatous component. These findings are consistent with prior reports: Perrone et al. demonstrated moderate‐to‐strong Trop‐2 staining in 47% of epithelial ovarian cancer tissues by IHC [15], and Lopez et al. reported strong/diffuse Trop‐2 staining in 30% of carcinosarcoma FFPE tumors, with expression predominantly confined to the carcinomatous component [16]. More recently, Moufarrij et al. detected Trop‐2 protein expression in ≥ 90% of primary uterine carcinosarcomas and similarly found higher expression in carcinomatous than sarcomatous components, while also demonstrating that SG retains preclinical efficacy even in tumors with heterogeneous Trop‐2 expression [27]—a mechanism of particular relevance to OCS given the differential expression between components observed in our cohort (carcinomatous component: 25%–90%, median 50%; sarcomatous component: low in 6 of 7 evaluable cases).

In genomic analysis, two cases showed WGD. WGD causes chromosomal duplication and amplification, is associated with aggressive tumor behavior, promotes cancer progression, and contributes to treatment resistance. In HGSC, WGD has been reported to be significantly more frequent in late‐stage disease, consistent with its acquisition during disease progression [23]. WGD has also been shown to be associated with *CCNE1* amplification, suggesting that OCS may be an aggressive subtype of *CCNE1*‐amplified HGSC. In this study, CS7, which had *CCNE1* amplification and WGD, resulted in death with an OS of 8.2 months, showing an extremely poor prognosis (Table 1, Figure 2). One out of nine cases was identified as Lynch syndrome and showed dMMR, a biomarker associated with sensitivity to immune checkpoint inhibitors. This Lynch syndrome case had a *TP53* mutation, but p53 immunohistochemical staining showed weak positivity and did not display the typical *TP53* mutation pattern staining. Our findings are consistent with a model in which HGSC undergoes EMT, although direct transcriptomic or functional evidence was not obtained in this study; some cases also appear to be related to Lynch syndrome.

Furthermore, in OCS, amplifications of several important genes including *CCND1*, *CCNE1*, *PRKCI*, *GNAS*, and *ERBB2* were observed (Figure 2). These genes play important roles in cell cycle control (cyclin D1, cyclin E1), signal transduction (PRKCI, GNAS), and receptor tyrosine kinase pathways (HER2). Amplification of these genes suggests potential therapeutic targets such as CDK4/6 inhibitors for *CCND1* and *CCNE1* amplified tumors, and anti‐HER2 therapy for *ERBB2*‐amplified cases. Cyclin D1 is associated with poor prognosis when highly expressed in ovarian cancer [23, 25], and may contribute to the aggressive nature of OCS. *GNAS* amplification has been reported to be a driver of EMT in OCS in previous reports [17], in which chr20 amplification including *GNAS* was identified in the sarcomatous component, and in this study, *GNAS* amplification was found in 37.5% (3/8) of OCS cases; in CS6 the amplification was confined to the sarcomatous component, consistent with that report. PRKCI has been reported to prevent CD8+ T cell infiltration into tumors in ovarian cancer [26]. *PRKCI* amplification was found in 37.5% of cases and is thought to be a factor in the immune desert nature of OCS. In this study, *ERBB2* amplification was found in one case (12.5%), suggesting that anti‐HER2 drugs should be considered for *ERBB2*‐amplified OCS.

This study has several limitations that should be acknowledged. First, the retrospective design and small sample size (*n* = 9, genomic data available for *n* = 8) limit statistical power and generalizability. The rarity of OCS makes prospective, large‐scale studies challenging. Second, the heterogeneity in treatment regimens across cases precludes definitive conclusions regarding the comparative efficacy of different therapeutic strategies. As complete macroscopic resection was achieved in all cases, the relatively favorable OS observed in this cohort may partly reflect selection bias, and the contribution of individual treatment components cannot be determined. Third, in CS7 and CS9, the carcinomatous and sarcomatous components were intimately admixed without a clearly definable boundary, making strict separation for IHC scoring not always feasible. Fourth, component‐resolved genomic data were obtained in only six of the nine cases: CS7 was sequenced in bulk because the two components were inseparably admixed, the sarcomatous component of CS8 yielded DNA of insufficient concentration for sequencing, and CS9 could not be sequenced at all. This limits the strength of the inferences that can be drawn regarding component‐shared alterations and the proposed EMT‐driven pathogenesis. Fifth, all sequencing was performed on tumor tissue only, without matched germline (normal) controls; germline variants were excluded using algorithmic filtering and population‐frequency databases, so somatic and germline variants cannot be definitively discriminated, and the germline contribution to the variants reported here (e.g., *BRCA2* and *PALB2*) cannot be determined. In CS1, the diagnosis of Lynch syndrome had been established clinically and by dedicated germline genetic testing performed independently of the present panel analysis. Sixth, functional validation of the identified genomic alterations was beyond the scope of this study, and the therapeutic implications of these findings remain to be confirmed in larger cohorts. Seventh, as a single‐center cohort, these findings may not be representative of the broader OCS population and require multi‐institutional validation. Eighth, the immune microenvironment was characterized using only two markers (CD8 and PD‐L1); accordingly, the immune‐cold designation should be interpreted with caution, and a broader immune panel is warranted. Ninth, the targeted panel does not capture genome‐wide structural events or genes outside the panel, so the genomic complexity of these tumors may be underestimated. Finally, marker–outcome associations in this study are exploratory and hypothesis‐generating given the limited sample size. Future studies should validate these findings in larger, multi‐institutional cohorts with matched germline sequencing, employ broader immune profiling beyond CD8 and PD‐L1 (including additional tumor‐infiltrating lymphocyte subsets and antigen‐presentation markers), evaluate Trop‐2–targeted antibody–drug conjugates such as sacituzumab govitecan in dedicated preclinical and clinical studies of OCS, and incorporate transcriptomic and functional analyses to directly test the proposed EMT‐driven pathogenesis.

In conclusion, while ovarian and fallopian tube carcinosarcomas are generally considered to have a poor prognosis, multimodal treatment including maximum tumor excision may lead to a favorable prognosis. OCS appears to be a tumor where HGSC undergoes EMT and is particularly associated with *CCNE1* amplification and WGD, which are related to homologous recombination‐proficient HGSC, but some cases appear to be dMMR. OCS appears predominantly immune cold in our cohort; immune checkpoint inhibitor monotherapy is therefore unlikely to benefit most patients, with the possible exception of dMMR cases, although this requires validation in larger series. Trop‐2 expression is high in OCS, especially in the carcinomatous component, and Trop‐2–targeted ADCs, which may exploit a bystander effect, represent a promising hypothesis that warrants dedicated preclinical and clinical evaluation in OCS.

## Author Contributions

**Tatsuyuki Chiyoda:** conceptualization, formal analysis, funding acquisition, investigation, resources, writing – original draft, writing – review and editing. **Ryutaro Kawano:** formal analysis, data curation. **Tomomi Sakamaki:** data curation, investigation, writing – original draft, writing – review and editing. **Reika Takamatsu:** formal analysis, data curation. **Kohei Nakamura:** data curation, formal analysis, methodology, writing – original draft. **Mio Takahashi:** writing – review and editing, resources. **Takuma Yoshimura:** writing – review and editing, resources. **Tomoko Yoshihama:** writing – review and editing, resources. **Kensuke Sakai:** writing – review and editing, resources. **Hiroshi Nishihara:** methodology, writing – review and editing. **Wataru Yamagami:** supervision, resources. **Shinya Oki:** writing – review and editing, resources. **Daisuke Ochiai:** resources, writing – review and editing. **Yumiko Kimura:** writing – review and editing, resources. **Mitsuyo Jisaka:** resources, writing – review and editing.

## Funding

This work was supported by JSPS KAKENHI (Grant‐in‐Aid for Scientific Research [B]) (Grant number 23K21475).

## Ethics Statement

This study was approved by the Ethics Committee of Keio University Hospital (approval numbers: 20070081 and 20190111). The study complied with the ethical standards of institutional and national research committees and adhered to the 1964 Declaration of Helsinki and its later amendments.

## Consent

Informed consent was given by patients for the use of their samples and information (Keio University Hospital approval number: 20070081). Patients were free to revoke their consent at any time point. In this study, only samples from patients who did not revoke their consent were used. All informed consent was obtained from the subjects.

## Conflicts of Interest

Tatsuyuki Chiyoda received a research grant from Takeda Pharmaceuticals Company Limited. Hiroshi Nishihara is an editorial board member of *Cancer Science*. The other authors declare no conflicts of interest.

## Data Availability

The data that support the findings of this study are available from the corresponding author upon reasonable request.

## References

[1] M. Kozłowski , K. Nowak , A. Kordek , and A. Cymbaluk‐Płoska , “Therapeutic Management of Rare Primary Ovarian Neoplasms: Carcinosarcoma, Leiomyosarcoma, Melanoma and Carcinoid,” International Journal of Environmental Research and Public Health 18 (2021): 18. 10.3390/ijerph18157819 PMC8345663 34360112

[2] M. A. Powell , V. L. Filiaci , M. L. Hensley , et al., “Randomized Phase III Trial of Paclitaxel and Carboplatin Versus Paclitaxel and Ifosfamide in Patients With Carcinosarcoma of the Uterus or Ovary: An NRG Oncology Trial,” Journal of Clinical Oncology: Official Journal of the American Society of Clinical Oncology 40 (2022): 968–977. 35007153 10.1200/JCO.21.02050 PMC8937015

[3] J. A. Rauh‐Hain , R. Gonzalez , A. J. Bregar , et al., “Patterns of Care, Predictors and Outcomes of Chemotherapy for Ovarian Carcinosarcoma: A National Cancer Database Analysis,” Gynecologic Oncology 142 (2016): 38–43. 27107722 10.1016/j.ygyno.2016.04.025

[4] S. Boussios , A. Karathanasi , N. Zakynthinakis‐Kyriakou , et al., “Ovarian Carcinosarcoma: Current Developments and Future Perspectives,” Critical Reviews in Oncology/Hematology 134 (2019): 46–55. 30771873 10.1016/j.critrevonc.2018.12.006

[5] T. Chiyoda , H. Tsuda , H. Tanaka , et al., “Expression Profiles of Carcinosarcoma of the Uterine Corpus‐Are These Similar to Carcinoma or Sarcoma?,” Genes, Chromosomes & Cancer 51 (2012): 229–239. 22072501 10.1002/gcc.20947

[6] R. L. Hollis , I. Croy , M. Churchman , et al., “Ovarian Carcinosarcoma Is a Distinct Form of Ovarian Cancer With Poorer Survival Compared to Tubo‐Ovarian High‐Grade Serous Carcinoma,” British Journal of Cancer 127 (2022): 1034–1042. 35715633 10.1038/s41416-022-01874-8 PMC9470739

[7] M. A. Powell , L. Bjørge , L. Willmott , et al., “Overall Survival in Patients With Endometrial Cancer Treated With Dostarlimab Plus Carboplatin‐Paclitaxel in the Randomized ENGOT‐EN6/GOG‐3031/RUBY Trial,” Annals of Oncology 35 (2024): 728–738. 38866180 10.1016/j.annonc.2024.05.546

[8] O. Gotoh , Y. Sugiyama , Y. Takazawa , et al., “Clinically Relevant Molecular Subtypes and Genomic Alteration‐Independent Differentiation in Gynecologic Carcinosarcoma,” Nature Communications 10 (2019): 4965. 10.1038/s41467-019-12985-x PMC6823358 31672974

[9] B. J. Monk , N. Colombo , A. M. Oza , et al., “Chemotherapy With or Without Avelumab Followed by Avelumab Maintenance Versus Chemotherapy Alone in Patients With Previously Untreated Epithelial Ovarian Cancer (JAVELIN Ovarian 100): An Open‐Label, Randomised, Phase 3 Trial,” Lancet Oncology 22 (2021): 1275–1289. 34363762 10.1016/S1470-2045(21)00342-9

[10] J. Ordner , J. M. Gutierrez Amezcua , A. Marcus , and P. S. Shukla , “Programmed Death Ligand 1 (PD‐L1) Expression and CD8 + Tumor‐Infiltrating Lymphocyte‐Based Tumor Immune Microenvironment Classification in Gynecologic Carcinosarcoma: Prognostic Impact and Implications for Therapy,” International Journal of Gynecological Pathology: Official Journal of the International Society of Gynecological Pathologists 42 (2023): 364–375. 35639400 10.1097/PGP.0000000000000890

[11] J. Zhu , H. Wen , X. Ju , R. Bi , W. Zuo , and X. Wu , “Clinical Significance of Programmed Death Ligand‐1 and Intra‐Tumoral CD8+ T Lymphocytes in Ovarian Carcinosarcoma,” PLoS One 12 (2017): e0170879. 28125702 10.1371/journal.pone.0170879 PMC5268655

[12] R. Raji , F. Guzzo , L. Carrara , et al., “Uterine and Ovarian Carcinosarcomas Overexpressing Trop‐2 Are Sensitive to hRS7, a Humanized Anti‐Trop‐2 Antibody,” Journal of Experimental & Clinical Cancer Research 30 (2011): 106. 22075385 10.1186/1756-9966-30-106 PMC3224774

[13] E. Bignotti , P. Todeschini , S. Calza , et al., “Trop‐2 Overexpression as an Independent Marker for Poor Overall Survival in Ovarian Carcinoma Patients,” European Journal of Cancer (Oxford, England: 1990) 46 (2010): 944–953. 20060709 10.1016/j.ejca.2009.12.019

[14] J. Tymon‐Rosario , M. Gorman , D. L. Richardson , C. Washington , and A. D. Santin , “Advances in Antibody‐Drug Conjugates for Gynecologic Malignancies,” Current Opinion in Obstetrics & Gynecology 35 (2023): 6–14. 36484278 10.1097/GCO.0000000000000838

[15] E. Perrone , S. Lopez , B. Zeybek , et al., “Preclinical Activity of Sacituzumab Govitecan, an Antibody‐Drug Conjugate Targeting Trophoblast Cell‐Surface Antigen 2 (Trop‐2) Linked to the Active Metabolite of Irinotecan (SN‐38), in Ovarian Cancer,” Frontiers in Oncology 10 (2020): 118. 32117765 10.3389/fonc.2020.00118 PMC7028697

[16] S. Lopez , E. Perrone , S. Bellone , et al., “Preclinical Activity of Sacituzumab Govitecan (IMMU‐132) in Uterine and Ovarian Carcinosarcomas,” Oncotarget 11 (2020): 560–570. 32082489 10.18632/oncotarget.27342 PMC7007291

[17] C. S. Herrington , A. J. Oswald , L. J. Stillie , I. Croy , M. Churchman , and R. L. Hollis , “Compartment‐Specific Multiomic Profiling Identifies SRC and GNAS as Candidate Drivers of Epithelial‐to‐Mesenchymal Transition in Ovarian Carcinosarcoma,” British Journal of Cancer 130 (2024): 327–335. 38097740 10.1038/s41416-023-02508-3 PMC10803731

[18] T. Yoshimura , K. Nakamura , T. Chiyoda , et al., “Next‐Generation Sequencing Outperforms Proactive Molecular Risk Classifier for Endometrial Cancer (ProMisE) in Endometrial Cancer Molecular Classification,” BJC Reports 3 (2025): 37. 40394155 10.1038/s44276-025-00145-2 PMC12092810

[19] K. Nakamura , E. Aimono , J. Oba , et al., “Estimating Copy Number Using Next‐Generation Sequencing to Determine ERBB2 Amplification Status,” Medical Oncology (Northwood, London, England) 38 (2021): 36. 33710417 10.1007/s12032-021-01482-1 PMC7954749

[20] M. Ogiri , R. Seishima , K. Nakamura , et al., “Real‐World Application of Next‐Generation Sequencing‐Based Test for Surgically Resectable Colorectal Cancer in Clinical Practice,” Future Oncology (London, England) 18 (2022): 2701–2711. 10.2217/fon-2022-0122 35818975

[21] G. Karpathiou , C. Chauleur , P. Dal Col , et al., “An Immunohistochemical Analysis of CD3, PD‐L1, and CTLA‐4 Expression in Carcinosarcomas of the Gynecological Tract and Their Metastases,” Pathology, Research and Practice 216 (2020): 153028. 32703493 10.1016/j.prp.2020.153028

[22] Y. K. Chae , M. Othus , S. P. Patel , et al., “SWOG/NCI Phase II Dual Anti‐CTLA‐4/PD‐1 Blockade in Rare Tumors: Nonepithelial Ovarian Cancer,” Clinical Cancer Research: An Official Journal of the American Association for Cancer Research 30 (2024): 5593–5600. 39417692 10.1158/1078-0432.CCR-24-0606 PMC11737520

[23] Z. Cheng , H. Mirza , D. P. Ennis , et al., “The Genomic Landscape of Early‐Stage Ovarian High‐Grade Serous Carcinoma,” Clinical Cancer Research: An Official Journal of the American Association for Cancer Research 28 (2022): 2911–2922. 35398881 10.1158/1078-0432.CCR-21-1643 PMC7612959

[24] S. J. Lee , J. G. Yoo , J. H. Kim , et al., “Gynecologic Oncology in 2024: Breakthrough Trials and Evolving Treatment Strategies for Cervical, Uterine Corpus, and Ovarian Cancers,” Journal of Gynecologic Oncology 36, no. 1 (2025): e72. 39900347 10.3802/jgo.2025.36.e72 PMC11790998

[25] L. L. Quan , J. Y. Liu , L. X. Qu , et al., “Expression of Cyclin D1 Gene in Ovarian Cancer and Effect of Silencing Its Expression on Ovarian Cancer Cells Based on the Oncomine Database,” Bioengineered 12 (2021): 9290–9300. 34806539 10.1080/21655979.2021.2000225 PMC8810081

[26] S. Sarkar , C. A. Bristow , P. Dey , et al., “PRKCI Promotes Immune Suppression in Ovarian Cancer,” Genes & Development 31 (2017): 1109–1121. 28698296 10.1101/gad.296640.117 PMC5538434

[27] S. Moufarrij , H. Dopeso , D. N. Brown , et al., “TROP2 Expression and Therapeutic Targeting in Uterine Carcinosarcoma,” Gynecologic Oncology 197 (2025): 129–138. 40344963 10.1016/j.ygyno.2025.04.590 PMC12160578

